# Chromoblastomycosis: New Perspective on Adjuvant Treatment with Acitretin

**DOI:** 10.3390/diseases11040162

**Published:** 2023-11-08

**Authors:** Walter Belda, Luiz Felipe Domingues Passero, Caroline Heleno Chagas de Carvalho, Paula Celeste Rubiano Mojica, Pablo Andrade Vale

**Affiliations:** 1 Dermatology Department, Medical School, University of São Paulo, Sao Paulo 05403-000, Brazil; caroline_heleno@hotmail.com (C.H.C.d.C.); paulacelesterubiano@gmail.com (P.C.R.M.); pablo.a.vale@gmail.com (P.A.V.); 2 Laboratory of Pathology of Infectious Diseases, Medical School, University of São Paulo, Sao Paulo 01246-000, Brazil; 3 Institute of Biosciences, São Paulo State University (UNESP), Sao Vicente 11330-900, Brazil; felipe.passero@unesp.br; 4 Institute for Advanced Studies of Ocean, São Paulo State University (UNESP), Sao Vicente 11350-011, Brazil

**Keywords:** chromoblastomycosis, treatment, acitretin, imiquimod

## Abstract

Chromoblastomycosis (CBM) is a neglected human disease, caused by different species of pigmented dematiaceous fungi that cause granulomatous and suppurative dermatosis. This infection is difficult to treat and there are limited therapeutic options, including terbinafine, itraconazole, and tioconazole. Classic treatment is administered for a long period of time, but some patients do not respond properly, and therefore, such therapeutic approaches possess low cure rates. Therefore, it is vital to develop new strategies for the treatment of CBM. In this regard, it has been observed that the association of immunomodulatory molecules such as glucan with therapy carried out with antifungal drugs improves cutaneous lesions in comparison to treatment with antifungal drugs alone, suggesting that drug association may be an interesting and significant approach to incorporate into CBM therapy. Thus, the aim of this work was to associate classical antifungal therapy with the adjuvants imiquimod and acitretin. In the present case, we reported a patient with extensive CBM caused by *Fonsaecae pedrosoi*, that affected an extensive area of the right leg, that was left without treatment for 11 years. He was treated with a classical combination of itraconazole and terbinafine via the oral route plus topical imiquimod and oral acitretin, as an adjuvant therapy. After five months of treatment, a significant regression of verrucous plaques was observed, suggesting that the use of these adjuvants combined with the classical antifungal drugs, intraconazole plus terbinafine, can reduce treatment time and rapidly improve the patient’s quality of life. This result confirms that the use of coadjuvant drugs may be effective in the treatment of this infectious disease.

## 1. Introduction

Chromoblastomycosis (CBM) is a chronic infectious, granulomatous, and suppurative mycosis caused by at least 39 different species of pigmented fungi, distributed in the following ten families of Ascomycota fillum: Chaetomiaceae, Cladosporiaceae, Didymellaceae, Dothioraceae, Herpotrichillaceae, Hysteriaceae, Microascaceae, Onygenacae, Pleosporaceae, and Pleurostomataceae [1,2].

In this context, the Herpotrichiellaceae family is highly relevant from an epidemiological perspective, since most human pathogens are included in it, such as *Fonsecaea* sp., *Cladophialophora carrionii*, and *Phialophora verrucosa*. Although CBM is spread throughout the world, its prevalence is significant in tropical and subtropical areas. Despite the importance of this infectious disease, CBM was only listed by the World Health Organization as a neglected disease in 2017 [3], considering that the majority of cases have been registered in low-income countries, affect people living in poor and remote areas, and only a few therapeutic options are available that are ineffective and cause side effects in patients.

In general, CBM affects people who work as farm laborers, vegetable harvesters, lumberjacks, and agricultural product traders. These people work with conventional harvest methods and are therefore in close contact with fungal species able to cause CBM [4], because these species exist in the soil, plants, thorns, and even in the buccal apparatus and stings of invertebrates. So, during a traumatic event with these materials, hyphae can be implanted into the skin, and after seven days, the differentiation of filamentous forms into muriform cells (or sclerotic cells) occurs in the cytoplasm of phagocytic cells [5]. This form is multiseptated, pigmented and has a globe-shape morphology. Furthermore, the presence of muriform cells is the expression of the etiologic agent in the tissue [6]. However, the incubation period is unknown, and the evolution of CBM is slow and progressive.

Frequently, a skin lesion appears at the site of hyphae inoculation and appears as an isolated macular lesion that develops into an erythematous papule, which over time increases in size and develops into a papulosquamous form. Skin lesions develop in extension and may be the consequence of scratching or autoinoculation of the satellite lesion. Furthermore, fungi can spread through tissues via the lymphatic system [7]. Depending on the immune response of the patient, the CBM develops into five different clinical forms: nodular, tumor type, verrucous, plaque, and cicatricial [6].

The nodular form is characterized by the presence of small nodules whose surface may be smooth, papillary, or scaly. If not properly treated, this clinical form gradually develops into the tumoral form, characterized by large lesions that present papillomatous morphology; furthermore, it can be more lobulated compared to the nodular clinical form and can exhibit tumor-like masses. The tumoral clinical form can be partially or completely recovered by epidermal remains, scabs, and black dots. In the verrucous clinical form, the lesions resemble warts; hyperkeratosis and black dots on the top of the skin lesions are remarkable features of such a clinical form of CBM. The plaque type is an unusual clinical form of CBM, and the lesions are planoconvex with various shapes and sizes, and the surface of the lesions is scaly. In the cicatricial form of the CBM, skin lesions develop via peripheral growth with atrophic scarring, while the middle of the lesion heals [8]. Some studies show that areas of the world have a prevalence of a specific clinical form, for example, in Central and South America, the verrucosa clinical form is the most prevalent form of CBM, with Cuba and Brazil being the most affected countries, respectively. On the African continent, the tumoral clinical form of CBM is the most common and Madagascar contributes significantly to the epidemiology of this clinical form. In Asia, the plaque form is the most prevalent, and India contributes with a significant number of cases of this clinical form [9].

Independent of the clinical form, at first the lesions are asymptomatic and patients normally develop all laboral activities; however, with the progress of the cutaneous disease, itching, accompanied or not by pain, has been reported, and such symptoms can interfere with normal activities [1,10]. Due to the morphology of CBM, a differential diagnosis must be performed and should be able to differentiate CBM from lobomycosis, protothecosis, verrucous tuberculosis, leishmaniasis, and leprosy, as well as non-infectious diseases such as squamous cell carcinoma and cutaneous sarcoidosis [11]. CBM has been considered a real challenge for physicians and patients because it is difficult to treat and has a low cure rate [12], despite the many attempts to develop an effective therapy. Furthermore, no guideline is available that recommends a gold standard therapy for CBM, or trials with a significant number of participants that suggest that a given drug or therapy is indicated for some specific clinical form. Furthermore, the few published trials report variable success rates, and some studies report relapses in up to 80% of patients [13].

In recent years, our research group has shown that alternative drugs can effectively treat complicated cases of CBM [14,15]. In this regard, imiquimod, an agonist of the Toll-like receptor 7/8 [16], administered alone or in combination with antimycotic drugs, was shown to be capable of improving CBM lesions in patients [14,17]. Furthermore, the combination of imiquimod with acitretin was successfully used in the treatment of CBM. Possibly, the efficacy of this adjuvant therapy is related to the immunomodulatory activity of imiquimod, which is a 7/8 Toll-like receptor agonist that can drive the immune response to a Th1 immune response [18]; this, in turn, should be considered an interesting adjuvant for the treatment of intracellular pathogens, such as CBM etiologic agents [19]. Additionally, the efficacy of this combined therapy is based on the fact that acitretin is an oral vitamin A derivative, capable of inhibiting epidermal proliferation [20], inflammatory processes, and angiogenesis. Therefore, it is believed that during treatment with antifungal drugs plus adjuvant therapy, acitretin acting as a keratoplastic agent allows the antimycotic drug to reach the site of cutaneous infection [21]; additionally, imiquimod can activate macrophages to microbicidal activity, which can potentialize the efficacy of the association.

Thus, based on our previous results, this case report demonstrates that the classical combination of the antimycotic drugs itraconazole and terbinafine administered along with the adjuvant drugs imiquimod and acitretin leads to a significant improvement in an extensive and severe lesion caused by *F. pedrosoi*; such results suggest that these adjuvants can be incorporated into the therapy of extensive lesions caused by the etiological agents of CBM.

## 2. Case Report

A 65-year-old farmer from Ceará state, Brazil, reported the appearance of verrucous lesions that started on the back and outer side of the right foot 11 years before seeking medical attention. Initially, the lesion appeared as a small nodule, without symptoms, but gradually extended along the leg and knee. As injuries did not prevent him from working as a farmer, he did not seek specialized medical care during the first 12 months after the appearance of the lesion. After this period of time, he sought medical assistance in his municipality. He was treated with topical neomycin and cephalexin (1000 mg/day) via the oral route for 30 days, associated with itraconazole 100 mg/day for 4 months; however, no improvements were observed. Due to the lack of effectiveness, the patient abandoned the medical follow-up.

He came to the “Ambulatório de Micoses Profundas do Hospital das Clínicas da Universidade de São Paulo” eleven years after the onset of the condition, exhibiting an extensive verrucous lesion that affected the back of the right foot, leg, and right thigh, that resembled chromoblastomycosis.

**Figure 1:**
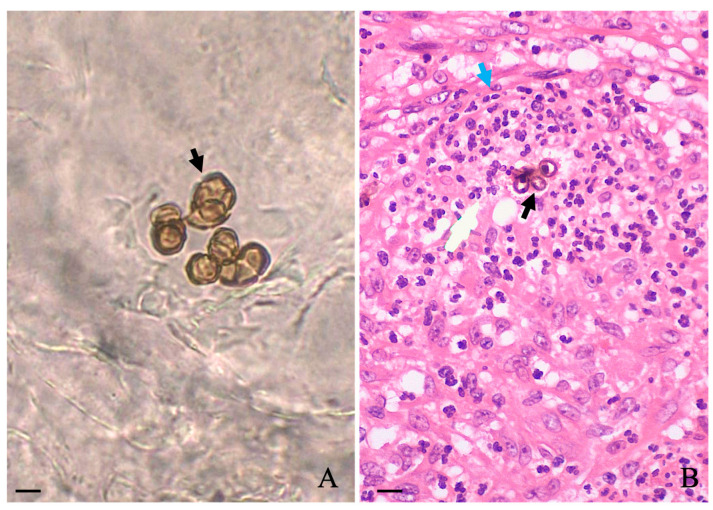
Microscopic observation of scales collected in three different regions of affected areas revealed the presence of muriform cells ((**A**); black arrow), which were also observed in the histological section of the skin stained with HE (black arrow) as observed in (**B**). Scale: 10 μm.

A slightly foul odor emanating from the lesions was noticed, and according to the patient, the lesion itched. During this period between the abandonment of therapy and the first hospital appointment, he stated that no topical or systemic therapy was used. Biochemical levels of blood glycemia, cholesterol, liver transaminases, gamma-glutamyl transferase, triglycerides, bilirubin, urea, and creatinine were normal. Additionally, the patient was serologically negative for hepatitis, syphilis, and HIV. Microscopic examination of the three different lesions in a 10% potassium hydroxide (KOH) wet mount revealed the presence of muriform cells in all samples (Figure 1A, black arrow). A histopathological study carried out in skin sections collected from the leg lesion and stained with hematoxylin and eosin (HE) showed pseudoepitheliomatous hyperplasia in the epidermis and muriform cells (black arrow) surrounded by granuloma in the dermis (Figure 1B, blue arrow).

Culture of the lesion smears, performed in Sabouraud dextrose agar medium, led to the morphological identification of *Fonsecaea* sp. after the 15th day of culture and microculture. In order to identify species, the colonies of the fungus were subjected to the MALDI-TOF MS analysis. A standard protein extraction was performed using ethanol and formic acid [22] and 1 μL aliquot of the sample was submitted to the MALDI-TOF MS analysis using a Vitek MS™ instrument (bioMérieux). For each acquisition group, a standard (*Escherichia coli* ATCC 8739) was included to calibrate the instrument and validate the run. Spectra were generated using LaunchpadTM v2.8 software (bioMérieux) and analyzed using SARAMIS Premium v4.11TM for Research Use Only (RUO) software SARAMIS Premium v4.11™ (bioMérieux). In this regard, the MALD-TOF MS analysis led to the identification of *Fonsecaea pedrosoi* as the etiologic agent with 99.9% confidence level values by comparing the obtained spectra with an in-house SuperSpectrum library HCFMUSP 02 [23].

**Figure 2:**
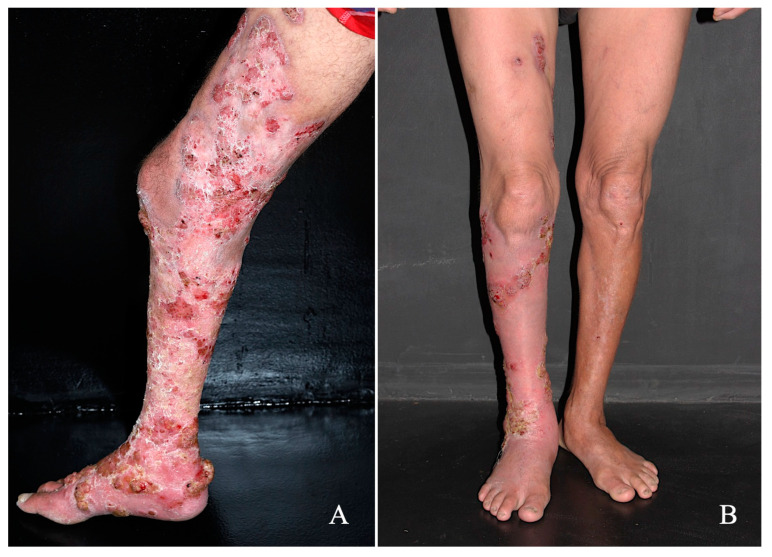
Patient with CBM exhibiting an extensive verrucous lesion that affected the back of the right foot, leg, and right thigh (**A**) was treated with the conventional antimycotic drugs itraconazole (200 mg/day) and terbinafine 250 mg/day, along with the coadjuvant drugs imiquimod once a day, five times a week by the topical route, and acitretin 50 mg/day; after five months of treatment, a significant improvement in the lesion was observed (**B**).

Treatment with the drug combination was started with itraconazole (200 mg/day) plus terbinafine (250 mg/day) along with the following co-adjutant drugs: topical 5% imiquimod once a day (Modik^®^), five times a week, and acitretin 50 mg/day. In comparison to the treatment onset (Figure 2A), after five months of therapy, a significant reduction in hyperkeratotic and verrucous plaques was observed; furthermore, in some areas, a complete disappearance of the lesions was recorded (Figure 2B). The patient no longer reported the presence of itching or the foul odor. During this treatment period, all biochemical parameters remained within normal limits and this patient is still being followed-up. Furthermore, no local adverse effects were observed during treatment.

## 3. Discussion

Since the description of the first case of CBM, different clinical forms have been described and a variety of pathogenic fungal species have been incriminated as etiologic agents of this disease; however, one of the most prevalent species is *F. pedrosoi* [24], the species incriminated as the etiologic agent of this patient.

Although studies have shown that these species are sensitive to classical antifungal drugs, such as terbinafine, itraconazole, and voriconazole [25], therapy in patients is a real challenge and these drugs must be associated with improved clinical symptoms in patients; however, in some cases, treatment is not efficient and other physical approaches are used in therapy to improve the quality of life of these patients, such as surgery, cryotherapy, and thermotherapy [11,26]. Despite the existence of different methods for treating CBM, to our knowledge, there are no randomized or comparative clinical trials suggesting which treatment would be the ideal for the different clinical forms of CBM [27].

In this context, in recent years, our research group has been looking for alternative methods to treat CBM, and by studying small case series, we observed that by combining some classical antifungal drugs such as itraconazol or terbinafine with immunomodulatory drugs glucan [28] or imiquimod [29], patients showed a significant improvement in the morphology of their lesions, increasing the cure rates. Furthermore, the use of topical imiquimod as a monotherapy also improved the lesions in a patient with CBM [14,30]. Other groups also demonstrated that combinations containing tioconazole, a conventional antimycotic drug, with acitretin as an alternative drug, lead to the disappearance of CMB lesions in a patient with psoriasis and CBM after 1 month of treatment [21]. Possibly, this combination was effective due to the different action mechanisms of both drugs: acitretin inhibits excessive cell growth and keratinization [31], reduces thickening of skin plaques, and tioconazole is an antifungal agent that ultimately inhibits ergosterol production [32]. Thus, by inhibiting the excessive development of plaques in the skin, acitretin may facilitate the passage of tioconazole to the place where the etiological agents of CBM parasitize, leading to their death.

Based on the study mentioned, we introduced the combination of antifungal drugs that are traditionally used in the treatment of CBM with imiquimod and acitretin, in extensive and aggressive cases of CBM that were unresponsive to classical treatment. Patients treated with such combinations presented a rapid and sustainable reduction in the appearance of large verrucous plaques, which facilitated and reduced the treatment time with classic antifungals. By reducing the time of classical treatment, the risk of side effects caused by prolonged use of these medications was reduced, as well as promoting a rapid improvement in the quality of life and self-esteem of these patients [15,21,33].

In the present case report, due to the duration, extension, and gravity of the CBM lesion, the classical association of antifungal drugs that consisted of terbinafine and itraconazole was maintained. Furthermore, the immunomodulator imiquimod along with acitretin was used as a combined therapy. In only five months of treatment, it was possible to identify a significant reduction in verrucous lesions in a rapid and sustainable manner, some of which even disappeared. Taking into account this drug association, it is possible that imiquimod activated phagocytes in a microbicidal state [34] and acitretin decreased plate thickness, allowing antimycotic drugs to access fungi in the skin. Also, it is important to note that both antimycotic drugs inhibit different enzymes, while itraconazole inhibits 14α-demethylase, and terbinafine inhibits squalene epoxidase [35,36]. Therefore, it is possible to build an ideal microenvironment to eliminate intracellular forms of *F. pedrosoi*. In fact, in only 5 months of treatment, a significant improvement was observed in the quality of life of this patient, allowing the patient to return to his family and social life, since even before treatment he isolated himself due to the grotesque appearance of the lesions and the bad odor they exhaled. Importantly, this association of drugs can be considered safe, as the patient did not show biochemical changes.

## 4. Conclusions

CBM remains a difficult-to-treat disease, and therapeutic failures are well documented. The introduction of immunomodulators into its treatment has shown good results. The introduction of acitretin and imiquimod as coadjuvant drugs, particularly in extensive cases, has proven to be an extremely important, effective, and safe ally, promoting a drastic and rapid reduction in the grotesque and verrucous appearance of this disease. Therefore, we believe that the use of acitretin and imiquimod as a coadjuvant therapy should be definitively incorporated into the therapeutic scheme of extensive cases such as the one reported here.

## Data Availability

The data presented in this study are available on request from the corresponding author.
